# Editorial: Synergic combination of natural bioactive compounds for preventing and treating human diseases

**DOI:** 10.3389/fnut.2025.1580609

**Published:** 2025-04-28

**Authors:** Maria Maisto, Anis Ben Hsouna, Gian Carlo Tenore

**Affiliations:** ^1^Department of Pharmacy, School of Medicine and Surgery, University of Naples Federico II, Naples, Italy; ^2^Department of Environmental Sciences and Nutrition, Higher Institute of Applied Sciences and Technology of Mahdia, University of Monastir-Tunisia, Mahdia, Tunisia

**Keywords:** nutraceuticals, synergistic effects, natural compounds, human health, functional food

**Figure 1:**
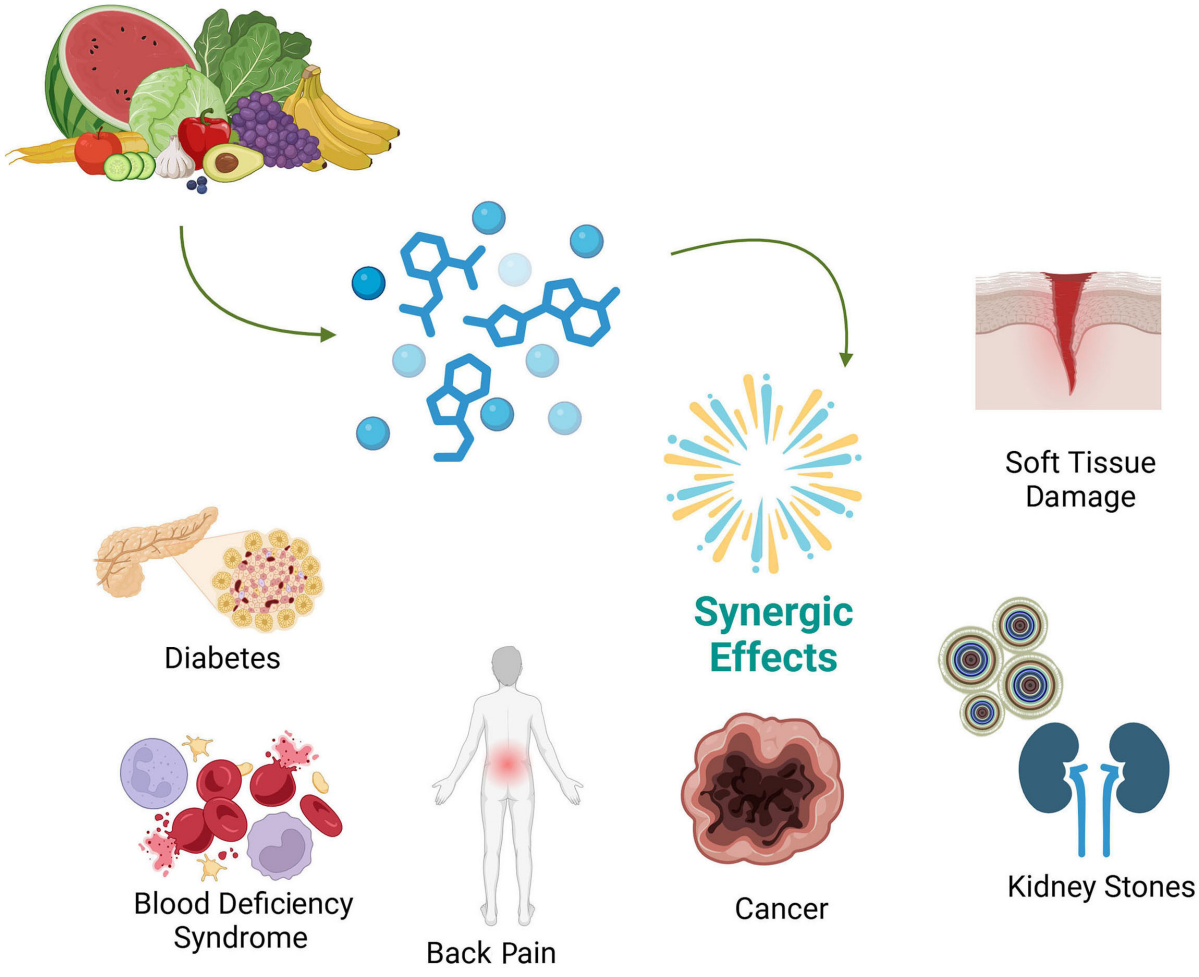
Synergic effects of natural bioactive compounds in the management of various human diseases.

In recent years, the focus on natural molecules and their potential to manage chronic diseases has grown exponentially (1–3), highlighting the complex interactions these substances have with biological pathways and their potential synergistic effects when used in combination (4). This editorial delves into the specifics of several key studies that have demonstrated the potential of natural compound combinations as alternative therapies for the treatment of different types of human diseases (Figure 1). Specifically, a recent study included in this Research Topic explored the combined effects of 3′-sialyl lactose and osteopontin, two human milk oligosaccharides, on influenza virus infection in an *in vitro* model of human laryngeal carcinoma cells (HEP-2). The study highlighted the reduction of pro-inflammatory cytokines, such as TNF-α and interleukin-6 (IL-6), suggesting that the synergy between these molecules may enhance the immune system's response to viral infection (Guo et al.).

In the area of metabolic diseases, a study recently published in the current collection focused on the role of diosgenin, extracted from fenugreek, in a diabetic rat model. The ability of diosgenin to modulate the diabetic state was investigated by evaluating its effects on the expression of the main molecular modulators implicated in glycemic control. Diosgenin appears to modulate the activity of GLUT4, facilitating increased glucose uptake into cells and affecting the insulin signaling cascade by activating IRS and PI3K, leading to the phosphorylation and activation of Akt, which in turn inhibits GSK-3β, resulting in enhanced glycogen synthesis (Tak et al.). Continuing in the context of diabetes, another study of this Research Topic investigated the effects of a multi-component formulation derived from ancient traditional medicine on the management of senile diabetic conditions. The authors find that the tested formulation, composed of a combination of 13 different plant-based extracts, can ameliorate diabetes by reducing islet cell apoptosis and resisting oxidative stress by regulating the insulin-mediated PI3K/AKT/GSK-3β pathway (Zhang Y. et al.). Continuing the discussion on diabetes, a recent review focused on the valuable synergic effects of the different bioactive molecules of *Phyllanthus emblica fruit* extract, highlighting that these compounds could potentially enhance diabetic treatment outcomes by leveraging their antioxidant and anti-inflammatory properties (Prananda et al.).

Moving to a completely different pathological setting, a comprehensive review investigated the role of vitamins A, C, D, and E in cancer prevention and therapy, delving into the multifaceted mechanisms by which these vitamins contribute to oncological health. By synthesizing data from a wide range of preclinical and clinical studies, the review highlighted how these vitamins act synergistically to combat cancer progression through several biological pathways. The authors described that, while vitamins C and E can provide a valuable defense against oxidative stress, vitamin A plays a crucial role in the epigenetic regulation of oncogenes and tumor suppressor gene expression, influencing cancer development and progression. Furthermore, vitamin D has been shown to modulate the inflammatory response by regulating cytokine production and inhibiting pathways that lead to inflammation. Based on such considerations, the authors suggest that the synergic combination of vitamins could be a useful multifunctional tool to prevent the progression of cancer (Guo et al.).

In line with this trend, other authors have investigated the effects of a nutraceutical formulation containing a blend of three standardized polyphenolic extracts- rosemary (*Rosmarinus officinalis* L.), ashwagandha (*Withania somnifera* L.), and sesame (*Sesamum indicum* L.) seeds- on the relief of back pain. The formulation was tested in a single-center, randomized double-blind study with three parallel arms. The study resulted in valuable benefits in improving health-related quality of life, mood, and sleep quality in the treated patients (Pérez-Piñero et al.). Other authors have also investigated the effects of a multicomponent formulation consisting of a mixture of various natural extracts, i.e., *Angelica sinensis* (Oliv.), *Carthamus tinctorius* L., *Saposhnikovia divaricata*, Schischk, g; *Arisaema erubescens*, and *Angelica dahurica* on the soft tissue injury. After only 10 days of oral treatment, a valuable pain reduction was observed in the treated group (Zhu H. et al.).

Another example of a synergistic combination of natural bioactive compounds is the combination of melatonin and palmitoylethanolamide in a nutraceutical formulation used for the prevention and treatment of immune disease. The results highlight that such combinations can reduce the release of immune-inflammatory modulators in the human mast cell line (HMC-1.2) at their bioaccessible concentration. Additionally, the nutraceutical formulation tested can reduce COX-2 mRNA transcription levels in stimulated HMC-1.2 and inhibit COX-2 enzymatic activity directly (Maisto et al.).

In contrast, other authors have examined the correlation between the Composite Dietary Antioxidant Index (CDAI) and the prevalence and recurrence of kidney stones. In the study, the CDAI was derived by standardizing dietary antioxidant intake from 24 h food recalls. The study assessed the prevalence and recurrence of kidney stones based on questionnaire responses. The association between the CDAI and both the prevalence and recurrence of kidney stones was investigated using multivariable logistic regression. The student results suggest that individuals in the top tertile had a 23% lower prevalence of kidney stones (OR = 0.77, 95% CI: 0.66, 0.90, *p* = 0.0011) and a 39% lower recurrence rate (OR = 0.61, 95% CI: 0.47, 0.80, *p* = 0.0003) than those in the bottom tertile. Finally, other studies have investigated the healthy effects of Catalpol (CA), derived from Rehmannia Radix, on blood deficiency syndrome (BDS), using 16S rRNA gene sequencing and metabolomic analysis of serum and spleen (Zhang W. et al.).

Another pivotal study investigated the impact of catalpol, a natural compound derived from *Rehmannia Radix*, on blood deficiency syndrome induced by chemotherapy drugs. This research demonstrated the efficacy of catalpol in mitigating the detrimental effects of these drugs on blood parameters in rats (Zhang W. et al.).

Finally, the multitarget activity of Ophiopogon D, a key natural organic compound found in *Ophiopogon japonicus*, to differentially modulate cardiovascular protection, immune modulation, anti-cancer, anti-atherosclerosis, anti-inflammatory properties, and efficacy against NAFLD (Non-Alcoholic Fatty Liver Disease), was investigated in a systematic review (Chen et al.).

In conclusion, the observed synergy between various natural bioactive compounds offers a promising frontier for the integrative management of health and disease. By harnessing the combined effects of these compounds, it is possible to enhance therapeutic outcomes and provide a basis for the development of novel treatment strategies that are both effective and sustainable. The future of medical treatment may very well depend on our ability to seamlessly integrate these natural elements with traditional medical practices, leading to holistic approaches that are not only preventive but also curative.
